# Non-pregnancy-related ovarian vein thrombosis: A rare cause of chronic abdominal pain

**DOI:** 10.5339/qmj.2021.13

**Published:** 2021-04-30

**Authors:** Shaikha D. Al-Shokri, Sundus Sardar, Fathima Shajeedha Ameerudeen, Mohammed Abdul Moqeeth

**Affiliations:** ^1^Department of Medicine, Hamad General Hospital, Doha, Qatar E-mail: s.alshokri@gmail.com; ^2^Weill Cornell Medicine Qatar, Doha; ^3^Department of Diagnostic Radiology, Hamad Medical Corporation, Doha, Qatar

**Keywords:** ovarian vein thrombosis, idiopathic, abdominal pain, right lower quadrant abdominal pain

## Abstract

Background: Ovarian vein thrombosis (OVT) commonly occurs during the peripartum and postpartum period. However, few cases of idiopathic OVT unrelated to pregnancy have been described.

Case report: We report a case of a previously healthy, 32-year-old female who presented with chronic right-sided abdominal pain. Abdominal and pelvic gadolinium-enhanced MRI showed a right OVT. The patient was not in the peripartum or postpartum period. Thrombophilia test results were negative, and no risk factors for thrombosis were noted. The patient received warfarin for 6 months, with resolution of her symptoms.

Conclusion: The presented case emphasizes the significance of considering OVT as a cause of unexplained abdominal pain in a young female. We describe a rare case of idiopathic OVT with a unique presentation.

## Background

Ovarian vein thrombosis (OVT) is a rare puerperal complications, with an incidence of about 1/600–1/2000 deliveries. It commonly occurs in the postpartum period. However, few cases have been reported in non-pregnant patients.^1,2^ Patients usually present with abdominal pain and fever. Diagnosis is confirmed using conventional radiological imaging, such as ultrasound, computed tomography (CT), and magnetic resonance imaging (MRI). Treatment of OVT includes anticoagulation therapy. In this report, we present a rare case of idiopathic OVT in a young female without risk factors for thrombosis. Informed consent for publication of this case was obtained from the patient.

## Case Report

A previously healthy 32-year-old Egyptian lady presented to the outpatient medical clinic with a 3-month history of right-sided abdominal pain exacerbated by movement and relieved with over-the-counter analgesics. The pain was related to food intake. She described the pain as stabbing in nature and radiating to the back. She denied any history of melena, dysphagia, weight loss, anorexia, nausea, and vomiting as well as history of fever or night sweats. She did not complain of vaginal discharge or menstrual abnormalities.

She is a nonsmoker and denied any personal or family history of venous thromboembolism. Her gynecological history was remarkable for five pregnancies. She had normal vaginal deliveries except for the last one, which required an emergency Cesarean section at 36 weeks and 5 days of gestation due to placenta previa type III. One month after delivery, the patient was started on desogestrel (0.075 mg oral daily) for contraception, which she continued for 2 years. Her menstrual period was irregular, and the last withdrawal bleeding was 2 weeks prior to clinic visit.

During the initial clinic visit, her vital signs were within normal limits (temperature, 36.7°C; heart rate, 78 bpm; blood pressure, 100/66 mmHg). Abdominal examination was significant for a mild tenderness in the right upper quadrant. Bowel sounds were noted.

Initial laboratory findings, including complete blood count, renal parameters, and electrolytes, liver function, lipase, and amylase levels, were within normal limits. Beta-hCG was negative. Initial differentials included cholecystitis, hepatitis, or hepatic abscess. Thrombosis of the portal vein is a possible differential diagnosis; however, in the absence of significant risk factors in the patient's history, further evaluation by ultrasound of the abdomen is required.

Abdominal ultrasound showed multiple hyperechoic focal lesions, the largest measuring 15 × 15 mm in the right hepatic lobe, suggestive of hemangiomas, indicating further evaluation by abdominal gadolinium-enhanced MRI, which confirmed hepatic hemangiomas; additionally, right OVT was suspected. Therefore, pelvic MRI with gadolinium-based contrast was requested, which revealed a prominent right ovarian vein measuring 8 mm in diameter with loss of flow void and persistent central nonenhancement with mild peripheral enhancement, indicating partial thrombosis of the right ovarian vein. Furthermore, the left ovarian vein was patent. Mild pelvic congestion and a small follicular cyst in the right ovary was noted (Figure 1A, 1B).

Further laboratory investigations were performed to evaluate the underlying cause of the thrombosis, including factor V Leiden mutation, ANA, lupus anticoagulant, anticardiolipin antibody, beta-2 microglobulin, and JAK2 mutation, which were all negative (Table 1). Few thrombophilia tests such as anti-thrombin III, Methylenetetrahydrofolate reductase (MTHFR) gene, and protein C were not performed.

## Management and Follow-up

The patient was started on anticoagulation (warfarin) for 6 months. The dose of warfarin was adjusted upon regular follow-ups in the anticoagulation clinic to maintain a target INR of 2–3. She was referred to an obstetrician and was advised to use barrier methods for contraception. Moreover, she was advised to avoid hormonal contraceptives due to the recent ovarian thrombosis and multiple hemangioma finding as oral contraceptives increase the size of hepatic hemangiomas and predispose transformation to malignant hepatomas.^28^ At her 6-month follow-up, she reported significant improvement of the abdominal pain. The patient was lost to follow-up as she traveled to her home country.

## Discussion

OVT is a rare but life-threatening complication that usually occurs in the intrapartum or postpartum period.^1,3,4^ The incidence of postpartum OVT is 1/600–1/2000 deliveries; the estimated incidence post-vaginal delivery is 0.18% and post Caesarian section is 2%.^1,2,5,6^ It was found that most OVT cases are diagnosed in the postpartum period due to the rise in estrogen hormone, causing a hypercoagulable status, or gravid uterine mechanical compression to the ovarian vein.^5^ Moreover, OVT may occur immediately after major pelvic surgeries.^5,7^

Most patients with underlying OVT usually present with fever, pelvic pain, abdominal mass, or abdominal pain.^8^ Moreover, OVT is commonly diagnosed within a few weeks postpartum.^1^ Symptomatic OVT is observed in 0.01%–0.05% of deliveries, mainly after Cesarean surgeries.^1^ Symptoms usually appear within 10 days post-delivery; however, it can present up to 4 weeks postpartum.^3^

In contrast, OVT cases may be asymptomatic. Few cases were detected during screening MRI, whereas others were reported in a setting of underlying malignancies, pelvic inflammatory disease (PID), or post-major pelvic surgeries.^1,9^

Previous studies showed that 50% of patients with OVT have other risk factors, such as prothrombotic conditions, including protein S deficiency, factor V Leiden mutation, and antiphospholipid syndrome.^3^ In the present case, the diagnosis of idiopathic OVT was established after ruling out hypercoagulable states, such as recent delivery, malignancies, major surgeries, and PID.^1^

There is a threefold increase risk of OVT in the peripartum and postpartum period due to reduced blood flow in the vein and stasis. Thrombosis tends to occur in the right gonadal vein due to its longer length, multiple incompetent valves, and the gravid uterine dextrorotation.^10,11^

Diagnosing OVT can be challenging, and laparotomy remains the gold standard in its diagnosis. Several studies have explored the role of different imaging methods as diagnostic tools^3^, and various studies have shown different results in the sensitivity and specificity of imaging modalities. For example, magnetic resonance (MR) angiography was found to have 100% specificity and 92% sensitivity in establishing an OVT diagnosis.^3,10^

CT scan with IV contrast has 78% and 62% sensitivity and specificity, respectively. CT scan is preferred over MR as it is time and cost-effective.^3,5,12,13^ Doppler ultrasound is commonly used due to its easy accessibility, lack of risk of radiation, and low cost. It has a sensitivity of 56% and a specificity of 42%.^3,12^ However, it is the least sensitive modality for the detection of OVT, as it cannot scan the whole length of the ovarian vein.^5,13^ Moreover, Doppler ultrasound is known to be operator-dependent, and bowel gases often result in poor-quality images.^14^

In rare cases, OVT can lead to lethal complications, including thrombus migration into the inferior vena cava (IVC), progression to pulmonary embolism (PE), and development of septic thrombophlebitis.^3^ Haris et al. reported that the mortality of PE as a result of untreated OVT is around 4% (6).

Moreover, the treatment of OVT remains debatable. There is no consensus in the international guidelines regarding the initiation, choice, and duration of anticoagulation. Few studies suggested conservative management, especially in incidentally detected thrombi in the postoperative period, unless complicated.^3,15^ However, most of the literature supports that OVT should be considered and managed as lower-extremity deep vein thrombosis (DVT) with regard to anticoagulation.^3^ Previous studies recommended treating thrombophlebitis-induced OVT with warfarin along with LMWH bridging for 7–10 days, combined with broad-spectrum antibiotics. Furthermore, when thrombus extends to the renal vein or IVC, warfarin should be continued (with LMWH bridging) for 3–6 months.^3^

Another area of controversy is surgical intervention in patients with OVT. Surgical options include IVC filter insertion or surgical ligation of the vein to prevent the extension of the thrombus. These are reserved for patients who failed or are unable to tolerate medical treatment.^5,16^ Additionally, patients with unstable thrombosis may be subject for surgical options.^2^

Our patient was diagnosed with idiopathic ovarian thrombosis. Her thrombophilia test findings were unremarkable, and she was not postpartum. The use of desogestrel (a progestin-only contraceptive) is less likely to contribute to the risk of developing thrombosis. Based on observational studies, there was no association between progestin-only pills and increased risk of venous thromboembolism (VTE) in the general population. She was started on LMWH bridging, followed by warfarin for 6 months. She was then referred to the hematology clinic, where no risk factors for thrombosis were identified. Her abdominal pain subsided, and she was well during the follow-up period.

## Conclusion

OVT is a common complication in the puerperal period; however, few cases have described idiopathic OVT in non-pregnant patients, and diagnosis and treatment in the latter group of patients remains controversial. The present case reveals the difficulty of the clinical diagnosis of OVT. It highlights the significance of OVT as a possible differential diagnosis in a non-pregnant female presenting with chronic abdominal pain.

### Abbreviations

OVT: Ovarian vein thrombosis

VTE: Venous thromboembolism

PE: Pulmonary embolism

DVT: Deep vein thrombosis

LMWH: Low-molecular-weight heparin

CT: Computed tomography

MRI: Magnetic resonance imaging

PID: Pelvic inflammatorydisease

### Acknowledgments

The authors would like to acknowledge Dr. Akram Twair (from the Radiology Department) for detecting the ovarian thrombus and helping in the diagnosis.

### Source(s) of financial support in the form of grants

None.

### Conflict of interest

The authors have no conflict of interest to declare.

### Author Contributions

SDA and SS: manuscript writing and literature review; FSAM: diagnostic image interpretation and reporting; MA: team supervisor and mentor, as well as manuscript review.

### Consent

Informed consent was obtained from the patient for the publication of this case report and any accompanying images.

### Ethics approval

The case was approved by Medical Research Center at Hamad Medical Corporation. (MRC # 04–20–100).

## Figures and Tables

**Figure 1a.**
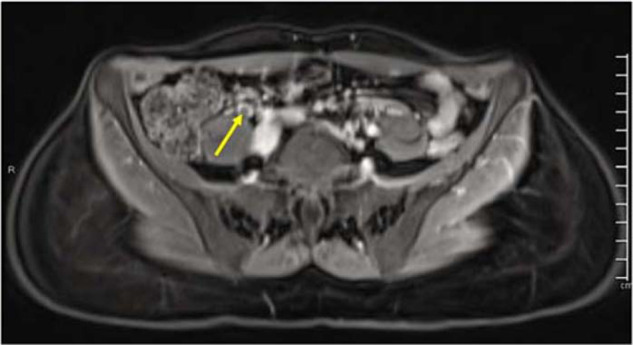
Post-contrast tbl1 fat-saturated sequence axial cut of the magnetic resonance (MR) scan of the pelvis demonstrates a prominent right gonadal vein with a partial filling defect, impressive of a thrombus.

**Figure 1b.**
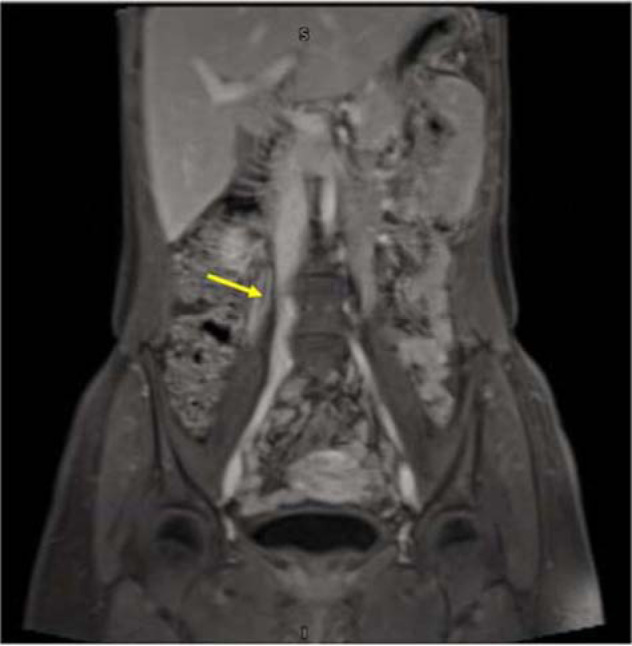
Post-contrast tbl1 fat-saturated sequence coronal cut of the abdominal MR shows a right ovarian vein thrombosis (arrow)

**Table 1:** Investigations table

Variable	Value	Normal value

ANA CTD screening	Negative	–

Factor V Leiden mutation	Negative	–

Lupus anticoagulant	34.2 seconds	30.4–45.3 seconds

Anti-cardiolipin IgG/IgM Ab	3.10GPL/2.30 MPL	Negative

Beta 2 microglobulin	1.42 mg/L	0.97–2.64 mg/L

JAK2 mutation	No evidence of V617F missense mutation within the JAK2 gene	–

B-hCG	< 0.5 mIU/mL	0.0–5.0 mIU/mL

AFP	2 IU/mL	0-6 IU/mL

